# Supplementary material for: Recovery coupling in multilayer networks
Source: Nat Commun. 2022 Feb 17;13:955. doi: 10.1038/s41467-022-28379-5 (PMC8854718; doi:10.1038/s41467-022-28379-5)
Supplement: Supplementary file 1 — Supplementary Information [file 41467_2022_28379_MOESM1_ESM.pdf]

Supplementary Information for Recovery Coupling of Multilayer Networks

Michael M. Danziger[1] and Albert-László Barabási[1]

[1] *Center for Complex Networks Research,*

Department of Physics, Northeastern University, Boston, MA

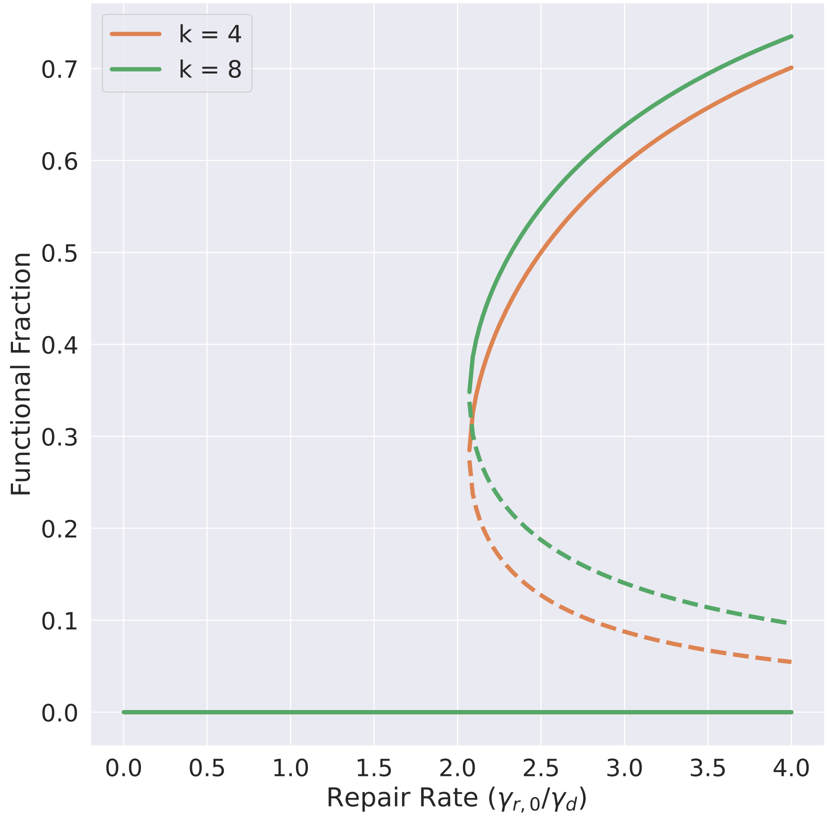

Supplementary Figure 1. Functional fraction of nodes for two non-symmetric recovery coupled networks. In this case the degree is different between the two networks but the ratio of $\gamma^{r,0}/\gamma_d$ is the same.

1. RECOVERY COUPLING FOR GENERAL PAIRS OF NETWORKS

In the main text we discussed the case of recovery coupling where the systems are symmetric. This reduces the two coupled equations to a single equation and simplifies the presentation. However, it is not necessary to assume that the networks are symmetric to solve the predictions of recovery coupling. The equations for a pair of networks would be

$$f_x = \frac{1}{1 + \frac{\gamma_x^{r,0}}{\gamma_x^d}(1 - \alpha_x(1 - u_y(1 - f_y)))} \quad (1)$$

$$f_y = \frac{1}{1 + \frac{\gamma_y^{r,0}}{\gamma_y^d}(1 - \alpha_y(1 - u_x(1 - f_x)))} \quad (2)$$

where for each network $i = x, y$, f_i is the fraction of non-functional nodes, $\gamma_i^{r,0}$ is the repair rate with full resource availability, γ_i^d is the damage rate, α_i is the coupling strength and

u_i is the function describing the number of connected nodes given that $1-f$ nodes are functional.

2. EFFECT OF LIMITED RESOURCES

We consider the effects of the damage on the repair resources by adopting the hypothesis that the repair rate is slowed down if resources are in use. Assuming that the full repair rate is achieved only when all of the resources are available and that the rate slows down as more resources are tapped, we consider a repair rate γ^r that is impacted by the amount of repair taking place, which is $\sim \gamma^r f$ according to the elastic assumption:

$$\gamma^r = \gamma_{r,0} - \alpha_R \gamma^r f + O\left((\gamma^r f)^2\right) \tag{3}$$

where we have parametrized the repair rate according to the amount of repair taking place in the system $\gamma^r f$, with α_R representing the first-order sensitivity to resource constraints. The precise value of α_R would be determined by the resource sensitivity of the recovery process and the resource capacity of the system, and is assumed to be > 0. Ignoring higher order terms, we obtain

$$\gamma^r = \frac{\gamma_{r,0}}{1+\alpha_R f}. \tag{4}$$

We note that the expected repair per unit time $\gamma^r f$ is monotonically increasing in f, indicating that under limited repair resources the amount of repair observed per unit time will rise less quickly than in the fully elastic case but will not ever decrese. In contrast, recovery coupling can lead to a decrease in repair per unit time with increasing f, as described in the main text and observed in the outage data.

We gain further insight by finding the fixed point:

$$f^* = \frac{1}{1+\frac{\gamma_{r,0}}{\gamma^d}\frac{1}{1+\alpha_R f^*}} \tag{5}$$

which, though quadratic, has only a single positive solution at:

$$f^* = \frac{\alpha_R - \frac{\gamma_{r,0}}{\gamma^d} + 1 + \sqrt{\left(\alpha_R - \frac{\gamma_{r,0}}{\gamma^d}+1\right)^2 + 4\alpha_R}}{2\alpha_R}. \tag{6}$$

This indicates that, though limited resources will indeed slow down the recovery, it cannot cause a collapse the way recovery coupling can.

3. MODELING DAMAGE PROCESSES

In the main text, we focused on the case where γ^d is a constant. This simplifies the expressions and enables us to focus on the main subject of the research, the recovery process. However, to assess whether we can expect to see the effects of recovery coupling in a given system, we need to make certain assumptions about the damage processes as well. For instance, if there are two coupled systems but during our observation period only one network has any damage at all, it is impossible in principle to detect the effects of recovery coupling.

If each node has a constant damage, as discussed in the main text, every moment in time will have $N_d \sim Poisson(N\gamma^d)$ outages added. The variance will be $N\gamma^d$ and we will not observe multiple orders of magnitude of damage. This also fails to capture the observed pattern of very low levels of damage most of the time, interspersed with high amounts of damage. When we look at the empirical distribution of new failures per 2 hour window we see that it more closely matches a Weibull distribution and that the autocorrelation is approximately 10x higher than the random case.

Furthermore, the correlated or uncorrelated nature of damage in the two networks can make a dramatic difference in terms of the system's resilience. As we show in Fig. 2, in the fully coupled case, a major shock affecting only one system does not severely impact the overall functionality. But a shock affecting both networks at the same time can trigger a system collapse.

When we model the damage process in both networks independently with a fat-tailed distribution like Weibull, we are able to recover the observed pattern of elastic behavior for small amounts of damage and inelastic behavior for large amounts.

4. THE OUTAGE OBSERVATORY

In recent years, electrical utilities have created live updating outage maps which list all of the current outages in their service area at a given time. The maps provide varying amounts of detail but all of them contain the exact location (latitude and longitude) and the number

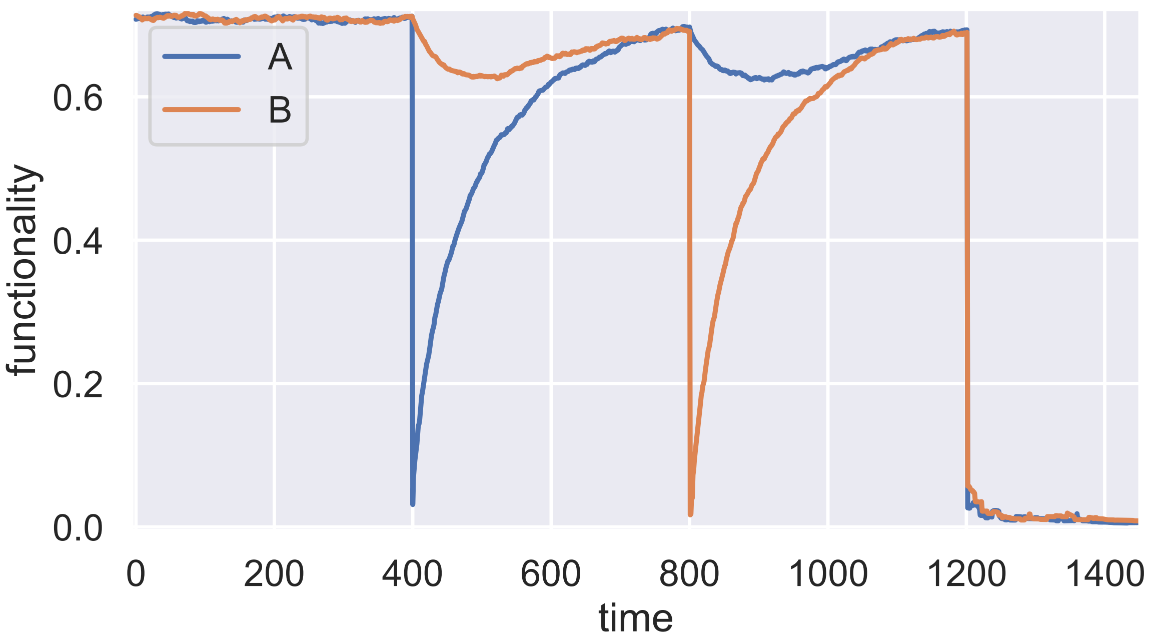

Supplementary Figure 2. When damage impacts networks A and B at different times, the system recovers with only minor functionality loss in the system that was not directly damaged. But when both networks undergo the large shock at the same time, the system of coupled networks collapses.

of customers affected. By scraping the outage map at regular intervals of approximately 10 minutes, we can determine when the outage was first reported and when it was repaired.

5. DETERMINING THE EXTENT OF THE ELASTIC REGION

We define elasticity in terms of the relation between marginal increase in outages in a system, compared to marginal increase in repairs in a system, each in a given unit of time. If that relation is constant, the system is said to display elastic behavior. The concept of elasticity has its origins in materials science where the input is deformation (strain) and the output is the restoring force (stress). To check for elasticity we measure, for each utility, the number of outages present in a given time window and the number of repairs executed during that same time window.

We use the concept of elasticity to quantitatively characterize the difference between normal and abonormal behavior, where the elastic behavior corresponds to normal, reseilient

Utility	Elastic Time Fraction	r^2_{max}	Inelastic Events Fraction	γ^r	Outages (2019 Total)	Outages (max at once)	Outage Duration (hours)	State
Centerpoint	0.9307	0.7908	0.4106	0.4700	464710	8653	2.8131	TX
PSEG	0.9668	0.9131	0.8989	0.5139	437744	36837	4.8902	NY
Entergy	0.9035	0.7488	0.4581	0.3920	424217	2930	3.9966	LA, TX, AL
PG&E	0.9126	0.8149	0.4058	0.4004	399143	4436	4.4653	CA
Duke	0.9457	0.9101	0.4803	0.4619	389987	10160	3.7078	NC, SC
FP&L	0.9910	0.9317	0.0386	0.4260	320392	1312	2.8666	FL
Ameren	0.9126	0.9238	0.4465	0.5954	310850	5625	2.0467	IL, MO
Oncor	0.9457	0.9478	0.2816	0.4894	308965	1492	1.9820	TX
Georgia Power	0.9668	0.8808	0.1836	0.5782	288041	4015	1.5874	GA
DTE	0.9789	0.7877	0.3025	0.2425	284365	6128	5.5773	MI
Eversource	0.9065	0.9360	0.7557	0.5690	242915	9234	4.2473	MA
Xcel	0.8824	0.8861	0.4476	0.5252	236576	4173	2.7228	MN, CO, NM
Dominion	0.9005	0.9035	0.5226	0.4726	227419	6990	3.0789	VA, NC
ComEd	0.9457	0.9105	0.5894	0.4796	213541	16185	3.3971	IL
First Energy	0.8734	0.9016	0.6062	0.4707	201032	7186	3.3019	PA
AEP	0.8884	0.8663	0.5897	0.4904	193102	6029	3.9269	TX
First Energy	0.9518	0.9198	0.4409	0.4213	189662	5800	3.1298	OH
Consumers	0.9095	0.8607	0.6891	0.4383	189566	6750	4.6479	MI
AEP	0.9578	0.9020	0.3423	0.4807	174472	2960	2.7568	OH
Appalachian	0.9246	0.7758	0.4140	0.3787	169888	2209	4.3629	VA, WV
First Energy	0.9578	0.8903	0.6534	0.3875	151465	7369	4.0280	NJ
Duke	0.9759	0.9105	0.1325	0.5039	129671	733	2.0469	FL
Swepco	0.8613	0.8632	0.6374	0.4818	107453	2904	3.9774	TX, LA
First Energy	0.9035	0.9054	0.6107	0.4816	102272	4535	3.4384	MD, WV
PECO	0.9849	0.8445	0.1831	0.3501	94125	1269	3.4093	PA
National Grid	0.9487	0.8839	0.4793	0.4377	85745	1660	3.6951	NY
ConEd	1.0000	0.8599	0.0040	0.3946	83482	1004	2.8571	NY
Rocky Mountain	1.0000	0.6333	0.0011	0.3315	80776	394	5.1317	UT, WY, ID
PSEG	0.9819	0.8836	0.3060	0.3605	78134	2015	3.5419	NJ
Midamerican	0.9759	0.9308	0.5285	0.6214	77156	4738	2.4717	IA, IL
PSO	0.9789	0.9424	0.1992	0.5865	76180	950	1.6580	OK
PPL	0.9216	0.8936	0.4708	0.5245	74922	1360	2.4128	PA
SCEG	0.9698	0.9215	0.4083	0.5786	72051	1713	1.9639	SC
Nipsco	1.0000	0.7016	0.0094	0.1687	68845	2721	6.6340	IN
WE	0.9578	0.9171	0.5310	0.5358	67416	1328	2.8353	WI
Kentucky	0.8553	0.8738	0.4265	0.5579	66590	424	1.8987	KY
SCE	1.0000	0.6934	0.0009	0.2863	66409	206	4.3118	CA
National Grid	0.9548	0.9103	0.7204	0.5366	66050	1961	4.8201	MA
Duke	0.9126	0.9045	0.5243	0.5623	64872	1131	2.3485	IN
Indiana Michigan	0.8884	0.8588	0.5966	0.5259	63964	1896	2.9670	IN, MI
Tampa	1.0000	0.9308	0.0061	0.5501	63471	763	1.2130	FL
Alliant	0.9578	0.9078	0.2735	0.5885	61600	456	1.9386	IA, WI
BGE	1.0000	0.8387	0.0037	0.2350	61219	679	3.8416	MD
NYSEG	0.9126	0.8690	0.6811	0.4430	56402	1758	4.2905	NY
Pacific	1.0000	0.4355	0.0016	0.1531	55072	394	6.9477	OR, WA
CMP	0.9276	0.8608	0.8421	0.4765	52735	4283	6.2879	ME
PSE	0.9879	0.8288	0.2901	0.3678	52390	1358	3.8333	WA
CPS	0.9970	0.9199	0.0678	0.5095	52158	578	1.4317	TX
PEPCO	0.9698	0.9353	0.1556	0.6064	51762	256	1.2669	DC
NV Energy	1.0000	0.9010	0.0034	0.7645	50257	759	1.1034	NV

Supplementary Table I. **Summary of Outage Observatory Observations** Elastic time fraction represents the fraction of the time that the system was observed to be in its elastic regime, as determined by the best linear fit as indicated by the r^2_{max} column. The inelastic events fraction column reports the fraction of total events which occur during the time that the system is not in the elastic regime. Though the time is comparatively short, it accounts for a disproportionate amount of the total events. Utilities with the same name are listed separately if they report outages in different states on different sites.

behavior because a consant fraction of outages is repaired per unit time. Inelastic bheavior, on the other hand, corresponds to a a lack of resilience because the fraction being repaired is reduced.

We measure elasticity by dividing the outage sequence into time windows and counting, for each time window, the number of outages and the number of repairs. Without exception in the utilitites observed, the response to small numbers of outages was elastic. To determine the point (if any) at which the elasticity breaks down, we divided the data by quantile and ran a linear regression on data from 0 to p for $p \in \{0, \ldots, 1\}$. We find that the slope is stable for most values of p and that r^2 steadily increases as more data is added to the fit. However, we detect a sharp drop-off in r^2 for many utilities, accompanied by a drop in γ^r (Fig. 3-4). We typically measure the drop at $p \approx 90$. We note that for some utilities, the drop does not occur at all and the linear fit remains valid for the entire domain (Table I). These utilities did not undergo a sufficiently large perturbation to exit the elastic regime during the observation period. This may be due to better repair capabilities or randomness in weather patterns.

While nearly 95% of the time, the repair time does not show a statistically significant deviation from the elastic response, when impacted by a large perturbation, the recovery of all utilities follows the same nonlinear response curve, offering empirical evidence of a remarkable universality in recovery. And though the utilities exhibit inelastic behavior only 5% of the observed time windows, nearly 25% of all observed outages occur within these time windows.

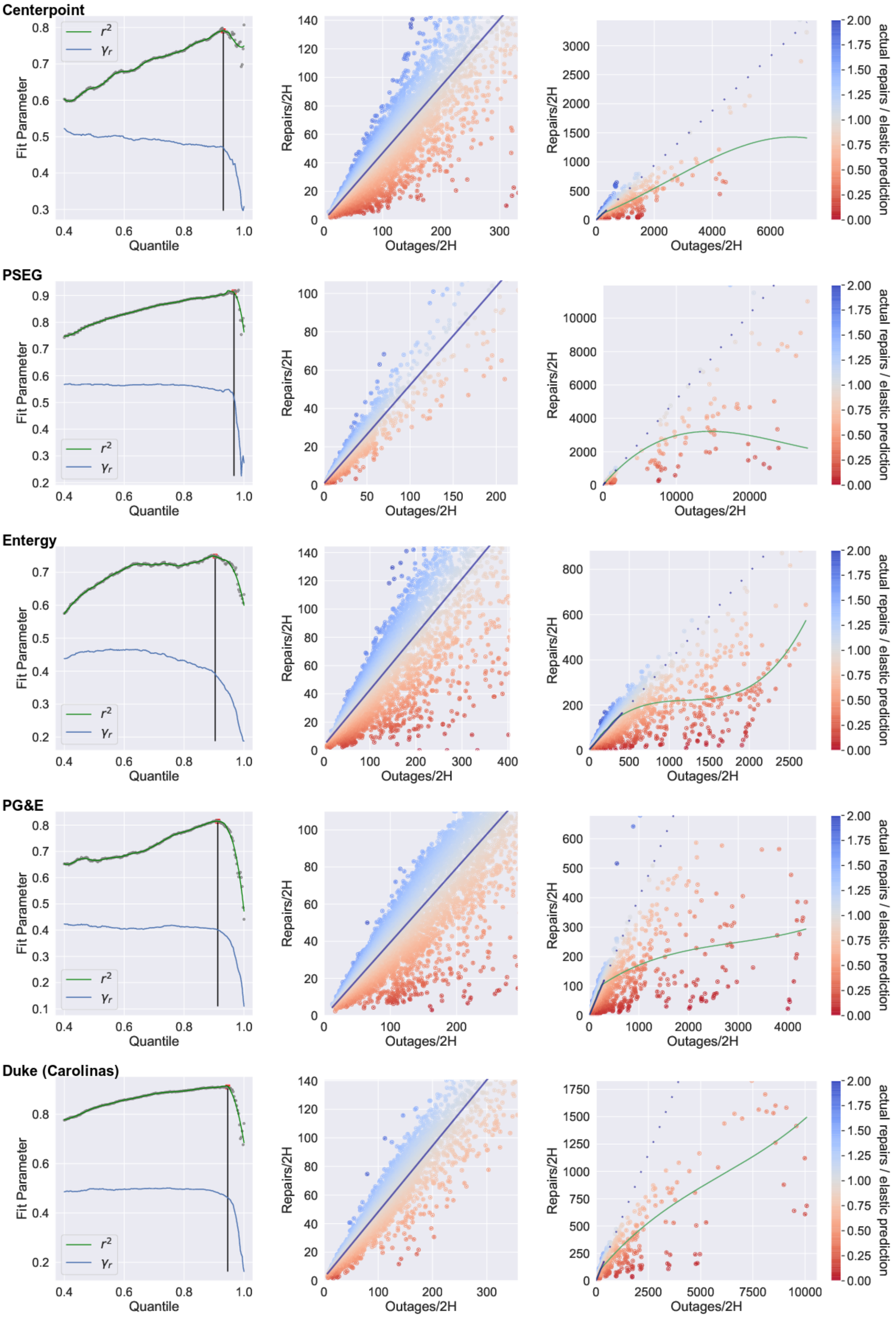

Supplementary Figure 3. **Calculation of elastic region (utilities 1-5)** (left) The fit parameters r^2 and γ_r (slope) as a function of the percentage of data included in the fit. (center) Outages and repairs for the region determined to be elastic. (right) Outages and repairs for the entire observed period. The blue line shows the elastic prediction, solid when in the elastic regime and dashed when not. To highlight the trend of the points we have added a spline fit in green. The five utilities shown here had the most total outages during the observed period.

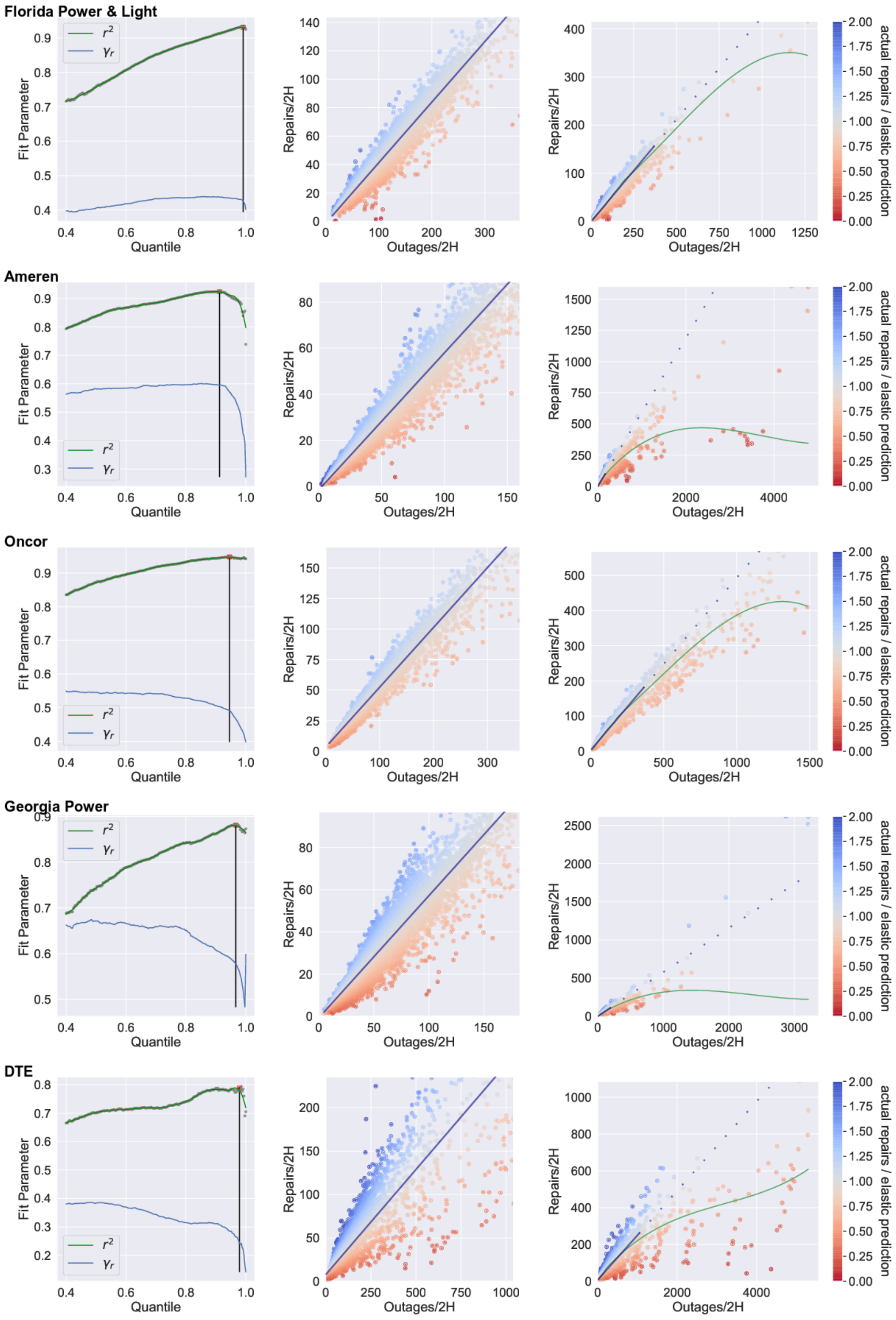

Supplementary Figure 4. **Calculation of elastic region (utilities 6-10)** (left) The fit parameters r^2 and γ_r (slope) as a function of the percentage of data included in the fit. (center) Outages and repairs for the region determined to be elastic. (right) Outages and repairs for the entire observed period. The blue line shows the elastic prediction, solid when in the elastic regime and dashed when not. To highlight the trend of the points we have added a spline fit in green. The five utilities shown here had the most total outages during the observed period, following those shown in Fig. 3.
